# Anatomical and Systemic Predictors of Early Response to Subthreshold Micropulse Laser in Diabetic Macular Edema: A Retrospective Cohort Study

**DOI:** 10.3390/jcm15030955

**Published:** 2026-01-24

**Authors:** Oscar Matteo Gagliardi, Giulia Gregori, Alessio Muzi, Lorenzo Mangoni, Veronica Mogetta, Jay Chhablani, Gregorio Pompucci, Clara Rizzo, Danilo Iannetta, Cesare Mariotti, Marco Lupidi

**Affiliations:** 1 Department of Sense Organs, Medicine and Dentistry Faculty, Sapienza University of Rome, Viale del Policlinico 155, 00161 Rome, Italy; oscarmatteo.gagliardi@uniroma1.it (O.M.G.);; 2 Eye Clinic, Department of Experimental and Clinical Medicine, Polytechnic University of Marche, 60126 Ancona, Italy; 3 Eye Clinic, Humanitas-Gradenigo Hospital, 10153 Torino, Italy; 4 Department of Ophthalmology, UPMC Eye Center, University of Pittsburgh, Pittsburgh, PA 15129, USA; 5 Eye Clinic, Department of Neurosciences, Psychology, Drug Research, and Child Health, University of Florence, AOU Careggi, 50139 Florence, Italy; 6 Fondazione Italiana Macula ETS, Di.N.O.G.Mi., University Eye Clinic, 16132 Genova, Italy

**Keywords:** biomarkers, diabetic macular edema, intraretinal fluid, subthreshold micropulse laser, AI segmentation, optical coherence tomography, diabetes mellitus

## Abstract

**Background/Objectives**: The aim of this study was to identify anatomical and systemic predictors of early (≤2 months) response to subthreshold micropulse laser (SMPL) in center-involving diabetic macular edema (DME) using automated AI-based OCT biomarker quantification. **Methods**: Retrospective observational study of 65 eyes. Spectral-domain optical coherence tomography (SD-OCT) volumes were analyzed with a CE-marked software (Ophthal v1.0; Mr. Doc s.r.l., Rome, Italy) to quantify intraretinal fluid (IRF) and subretinal fluid (SRF) volumes and outer retinal integrity (external limiting membrane, ELM; ellipsoid zone, EZ). SMPL (577 nm; 5% duty cycle; 200 ms; 150 µm; 250 mW) was applied in a high-density macular grid, sparing the foveal avascular zone. The primary endpoint was absolute and percentage change in IRF volume from baseline to follow-up; predictors of %IRF reduction were assessed by multivariable linear regression. **Results**: At 52 days (IQR 41–60), best-corrected visual acuity improved from 0.22 to 0.15 logMAR (*p* < 0.001). IRF volume decreased (median −0.045 mm^3^; *p* = 0.034) despite stable central subfield thickness. All eyes with baseline SRF (n = 5; median 0.026 mm^3^ [0.020–0.046]) achieved complete SRF resolution. Treatment-naïve eyes had greater %IRF reduction than pretreated eyes (59.6% vs. 11.5%; *p* = 0.029). High responders showed shorter diabetes duration than low responders (14.5 vs. 17 years; *p* = 0.025); however, treatment-naïve status was the strongest independent predictor of %IRF reduction (*p* = 0.028). **Conclusions**: AI-derived fluid volumetrics capture early SMPL response despite unchanged thickness. Treatment-naïve status and shorter diabetes duration may define a metabolic window for optimal early response in DME.

## 1. Introduction

Diabetic macular edema (DME) remains a leading cause of visual impairment in working-age adults [1], imposing a substantial burden on quality of life and healthcare systems [2]. Current clinical guidelines primarily rely on best-corrected visual acuity (BCVA) and OCT-derived central subfield thickness (CST) to guide management [3,4]. However, recent updates in multimodal imaging have highlighted a broad spectrum of structural biomarkers, supporting a biomarker-driven approach beyond BCVA and CST [5,6,7]. AI-assisted analyses have further emphasized this heterogeneity by identifying distinct OCT-based clinical phenotypes in DME cohorts [8].

While intravitreal anti-vascular endothelial growth factor (anti-VEGF) treatment is considered a primary approach in DME, it is associated with significant psychological [9] and financial pressure on patients [10], especially in high-income settings [11]. In addition, the short half-life [12], the residual risks of endophthalmitis [13], and systemic adverse events [14] of anti-VEGF agents limit long-term adherence.

In this context, subthreshold micropulse laser (SMPL) has emerged as a safe alternative to anti-VEGF in center-involving DME with CST < 400 µm [4]. SMPL delivers high-frequency, low-duty-cycle pulses targeting the retinal pigment epithelium (RPE) while sparing the neurosensory retina. It reduces vitreous inflammatory cytokines [15] and improves microvascular parameters, including a reduction in the superficial capillary plexus FAZ area [16]. This is biologically plausible, as inflammation is increasingly recognized as a key contributor to the pathophysiology of DME and its treatment response [17]. In the real world, SMPL treatment showed better visual and anatomical results than photocoagulation laser [18] and was comparable to anti-VEGF [19]. In addition, recent meta-analysis confirmed that SMPL decreases macular edema and reduces injection burden [20]. However, responsiveness to SMPL is highly variable.

A critical challenge in clinical practice is assessing the early response of DME to SMPL. Traditional metrics, particularly CST, are standard for monitoring long-term outcomes, yet they are often insufficient for detecting early response (2–3 months) [19,21,22]. Due to its one-dimensional nature, CST may mask early fluid reabsorption if overall retinal thickness remains stable. This limitation has significant clinical implications: the absence of early CST reduction may lead to the erroneous discontinuation of laser treatment, perceived as “ineffective”, resulting in premature switches to intravitreal injections and overtreatment.

Moreover, relying solely on thickness neglects the complexity of visual recovery. In DME, the BCVA is not solely determined by retinal fluid but also depends on the integrity of specific retinal microstructures at the fovea. For instance, visual prognosis is associated with hyperreflective foci (HRF) [5,6] and outer retinal integrity markers, including the ellipsoid zone (EZ) and external limiting membrane (ELM) [23]. In DME patients, EZ reflectivity has shown a consistent association with BCVA, persisting up to 5 years [24]. Likewise, baseline ELM integrity (%) has been identified as a relevant predictor of visual outcomes [25]. Notably, anti-VEGF agents showed to partially restore EZ and ELM [26]; however, evidence about SMPL is lacking.

On the other hand, evidence regarding the predictors of early SMPL response is scarce [19,22]. While fluid morphology (e.g., subretinal fluid) [27] and quantity are known to influence outcomes [28,29], the impact of systemic metabolic factors remains underexplored. In fact, tissue recovery seems not to occur in isolation: metabolic factors, such as chronically elevated HbA1c and the duration of diabetes, may further modulate the retinal reparative capacity and influence the visual acuity in DME [25,30,31]. Early identification of non-responders is crucial, as they may benefit from alternative therapies. However, the manual segmentation of OCT features is impractical, time-consuming, and prone to inter-observer variability, triggering the development of automated protocols [32]. In DME, the automatic quantification of fluid through AI algorithms has already been applied to evaluate the associations between anti-VEGF treatment and SRF and IRF [33], as well as to distinguish different subtypes of DME [34]. Still, it has never been investigated in the context of SMPL.

In this study, we aimed to characterize the anatomical and systemic predictors of early (≤2 months) SMPL response. Using an automated deep learning model previously validated for DME [35], we integrated volumetric quantification of retinal fluid and outer retinal integrity with systemic variables (HbA1c, diabetes duration, ocular history) to provide the first AI-based characterization of early SMPL responsiveness.

## 2. Materials and Methods

### 2.1. Study Design and Population

This retrospective observational study included patients with center-involving diabetic macular edema (DME), treated with subthreshold micropulse laser (SMPL) at Eye Clinic of the Polytechnic University of Marche, Ancona, Italy, between July 2023 and August 2025. The study adhered to the tenets of the Declaration of Helsinki. Given the retrospective design, no a priori sample size calculation was performed. Before the analyses, when two eyes were available, only the right eye was considered. All eligible consecutive cases were included.

Post-treatment OCT examinations were obtained according to real-world clinical scheduling, typically ranging from 6 to 9 weeks after treatment, depending on appointment availability. Accordingly, all high-quality OCT scans performed within this real-world timeframe were included. Images were reviewed to control the reliability of segmentation. This time window corresponds to the established period during which SMPL-induced modulation of RPE activity and retinal homeostasis is expected to manifest.

Inclusion criteria were as follows: (1) a diagnosis of type 1 or type 2 diabetes mellitus; (2) presence of center-involving DME suitable for SMPL treatment based on clinical judgment; and (3) availability of high-quality spectral-domain optical coherence tomography (SD-OCT) scans at baseline and post-treatment (≤2 months). Specifically, a minimum interval of 4 weeks after treatment was required for the post-treatment OCT examination. Exclusion criteria included significant media opacities preventing high-quality imaging (signal strength < 25), concomitant retinal diseases (e.g., AMD, vascular occlusions), history of vitreoretinal surgery, IVT treatment within 4 months before baseline, or treatment administered between SMPL treatment and the follow-up visit.

### 2.2. Image Acquisition and AI-Based Segmentation

All subjects underwent SD-OCT imaging using the Spectralis HRA + OCT2 platform (Heidelberg Engineering, Heidelberg, Germany). OCT volumes were exported and processed using Ophthal v1.0 (Mr. Doc s.r.l., Rome, Italy), CE-marked software implementing a previously validated deep learning algorithm for DME biomarker quantification [35].

The AI algorithm of Ophthal is based on Generative Adversarial Networks, falling under the category of semi-supervised learning. Specifically, this architecture leverages a combination of labeled data (manually segmented by expert clinicians) and a large volume of unlabeled data to generate a fully labeled dataset. Through a self-training mechanism, the model propagates labels across the database and learns to predict potential variations or image noise, thereby enabling effective and robust segmentation in real-world clinical scenarios.

The platform provides automated volumetric segmentation and quantification of OCT biomarkers, including intraretinal fluid (IRF) and subretinal fluid (SRF), and metrics of outer retinal integrity (ELM/EZ), without manual intervention. All segmentations were reviewed for plausibility, and scans not meeting predefined quality criteria were excluded. In accordance with the validation protocol of the AI software used [35], the acquisition protocol included a 6 × 6 mm volumetric map (49 B-scans) acquired in high-speed (HS) mode with an automatic real-time (ART) mean > 12 and quality index > 28.

For each eye, the following AI-derived metrics were extracted at baseline and at follow-up:

1. Fluid Volumetrics: Total volume (mm^3^) of intraretinal fluid (IRF) and subretinal fluid (SRF) within the 6 × 6 macular grid.
2. Hyperreflective Foci (HRF): Automatic count of hyperreflective foci (HRF) within the central 3 mm of the linear HR scan.
3. Outer Retinal Integrity: Percentage of disruption of the external limiting membrane (ELM) and ellipsoid zone (EZ) within the central 1 mm of the fovea, at 3 mm, and 6 mm, respectively.
4. Central Subfield Thickness (CST): defined as the mean retinal thickness within the central 1 mm ETDRS subfield, was extracted from the automated macular map of the OCT device (Heidelberg Engineering, Heidelberg, Germany).

### 2.3. Systemic and Clinical Data Collection

To evaluate the interaction between local anatomical features and systemic metabolic status, the following clinical variables were collected from electronic health records:

- Demographics: Age, gender.
- Metabolic Control: HbA1c levels (most recent value within 3 months of treatment), type of diabetes, and disease duration.
- Therapeutic History: Previous panretinal photocoagulation (PRP), number of prior intravitreal anti-VEGF injections, and time since the last injection.

### 2.4. SMPL Treatment Protocol

SMPL treatment was performed using a 577 nm yellow laser system. The laser parameters were set as follows: 5% duty cycle, 200 ms duration, spot size 150 µm, with no spacing between laser spots, according to Subthreshold Ophthalmic Laser Society guidelines [36]. Power was set to a fixed parameter of 250 mW, as recommended by the International Retinal Laser Society [37]. A high-density grid pattern was applied over the whole macular area, sparing the foveal avascular zone.

### 2.5. Outcomes

The primary outcome was the anatomical response to SMPL, assessed as the change in absolute and relative intraretinal fluid (IRF) volume on AI-based OCT segmentation between baseline and follow-up.

Secondary endpoints included the following:

1. Change in best-corrected visual acuity (BCVA);
2. Change in absolute and relative subretinal fluid (SRF) volume;
3. Change in hyperreflective foci (HRF) count;
4. Change in central subfield thickness (CST), automatically derived from the Early Treatment Diabetic Retinopathy Study ETDRS grid of the OCT instrument;
5. Changes in outer retinal integrity (ELM and EZ disruption) evaluated at 1, 3, and 6 mm.

### 2.6. Statistical Analysis

Descriptive statistics were used to summarize baseline demographics and AI-derived metrics. Continuous variables were tested for normality using the Shapiro–Wilk test. Normally distributed variables were summarized as mean ± standard deviation, whereas non-normal variables were reported as median and interquartile range (IQR). Categorical variables were described as counts and percentages.

To evaluate the effect of SMPL, paired comparisons between baseline and follow-up measurements were performed using paired *t*-tests for normally distributed variables and Wilcoxon signed-rank tests for non-normally distributed variables. Between-group comparisons (pretreated vs. naïve eyes) used independent *t*-tests or Wilcoxon rank-sum tests, as appropriate. Differences in categorical paired outcomes (e.g., SRF presence) were assessed using McNemar’s test. Correlations between anatomical changes and clinical variables (age, diabetes duration, number of prior injections) were evaluated using Spearman’s rank correlation. Multivariable linear regression models were used to assess predictors of IRF reduction (absolute and percentual), adjusting for age and prior anti-VEGF treatment. A sensitivity analysis was conducted by dividing eyes into high and low responders based on the median percentual IRF change, and diabetes duration was compared between groups using the Wilcoxon rank-sum test.

All tests were two-sided, and a *p*-value < 0.05 was considered statistically significant. Statistical analyses were performed using R software (v4.5.1).

## 3. Results

### 3.1. Baseline Demographic and Systemic Characteristics

A total of 65 eyes from 65 patients with center-involving DME treated with SMPL were included. The mean age at treatment was 70.0 ± 10.3 years; 41/65 patients (63.1%) were male. Most subjects had type 2 diabetes (64/65, 98.5%). The median duration of diabetes was 15 years (IQR 12–20). Median HbA1c was 6.4% (IQR 6.3–6.7). In total, 92.3% of the patients had HbA1c values below 7.0%.

Regarding systemic therapy, sixty patients (92.3%) were treated with oral hypoglycemic agents, two (3.1%) with insulin only, and three (4.6%) with combined therapy. Patient age and diabetes duration were not correlated in our cohort (ρ = 0.14, *p* = 0.24), allowing for independent assessment.

### 3.2. Ocular History

Prior intravitreal therapy (IVT) had been administered in 51/65 eyes (78.5%), whereas 14/65 eyes (21.5%) were treatment-naïve. The median number of IVTs before SMPL was 3 (IQR 5). Among pretreated eyes, the last administered drug was Bevacizumab in 29 eyes (56.9%), dexamethasone implant in 16 eyes (31.4%), and aflibercept or ranibizumab in 6 eyes (11.7%). Previous panretinal photocoagulation (PRP) had been performed in 42/65 eyes (64.6%).

### 3.3. Safety

SMPL treatment was well tolerated, with no laser-related adverse events (e.g., retinal burns, RPE damage, foveal structural alterations) observed in any patient.

### 3.4. Baseline Anatomical Characteristics

At baseline, median central subfield thickness (CST) was 342 µm (IQR: 307–392). Intraretinal fluid (IRF) was present in all eyes (100%), with a median absolute IRF volume of 0.326 mm^3^ (IQR: 0.135–1.091). Subretinal fluid (SRF) was detected in five eyes (7.7%; median 0.026 mm^3^; IQR: 0.020–0.046). At baseline, CST showed a moderate positive correlation with IRF volume (ρ = 0.40, *p* < 0.001) but was not significantly associated with BCVA (ρ = 0.19, *p* = 0.13). Hyperreflective foci (HRF) were observed in all eyes, with a mean count of 75.1 ± 28.5.

### 3.5. Visual Acuity Outcomes

In the overall cohort, BCVA remained stable or improved in 61/65 eyes (93.8%). No eyes showed a clinically meaningful loss (≥0.3 logMAR). The median BCVA improved from logMAR 0.22 (IQR: 0.15–0.30) to 0.15 (IQR: 0.10–0.30) (95% CI: 0.08 to 0.14; *p* < 0.001, Wilcoxon signed-rank test). Spearman’s rank correlation analysis revealed no significant association between ΔBCVA and either absolute IRF reduction (ρ = −0.135; *p* = 0.282) or % IRF reduction (ρ = −0.122; *p* = 0.332).

Stratification by baseline BCVA showed that significant visual improvement was achieved in both subgroups, although with different magnitudes. Patients with worse baseline BCVA (≥0.3 logMAR; n = 25) demonstrated higher gains, with a median BCVA of −0.125 (IQR: −0.176 to 0; *p* = 0.002, Wilcoxon signed-rank test). Patients with better baseline BCVA (<0.3 logMAR; n = 40) also showed a statistically significant improvement (*p* < 0.001, Wilcoxon signed-rank test); however, the median change was 0 (IQR: −0.058 to 0).

### 3.6. Predictors of Visual Response

**Figure 1:**
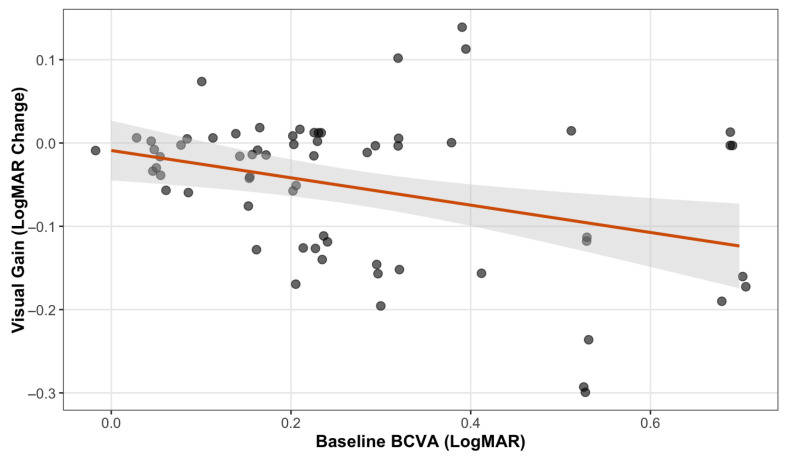
Correlation between baseline visual acuity and visual gain.

A significant correlation was observed between baseline BCVA and the magnitude of visual recovery. Univariate analysis showed that eyes with worse baseline visual acuity (higher logMAR scores) achieved greater visual gains compared to those with better initial vision, consistent with a ceiling effect (*p* = 0.004) (Figure 1).

The scatterplot illustrates the relationship between baseline BCVA (logMAR) and the visual outcome. To visualize overlapping data points, a slight jitter was applied. The red line represents the unadjusted linear trend. Multivariate analysis confirmed that baseline BCVA was the only significant predictor of visual improvement (*p* = 0.008), indicating that eyes with worse baseline visual acuity achieved greater functional recovery (ceiling effect).

To confirm this finding, a multivariate linear regression analysis was performed, adjusting for age, diabetes duration, HbA1c, and treatment status (naïve vs. pretreated). The model (R^2^ = 0.18; *p* = 0.039) identified BCVA as the sole significant independent predictor of visual improvement (β = −0.15; *p* = 0.008). Conversely, systemic and ocular factors did not significantly influence the visual outcome, including age (*p* = 0.87), diabetes duration (*p* = 0.29), metabolic control (HbA1c, *p* = 0.11), and treatment status (naïve vs. pretreated, *p* = 0.73).

### 3.7. Anatomical Outcomes

SMPL treatment induced a statistically significant reduction in intraretinal fluid (IRF). Specifically, median absolute IRF volumes decreased from 0.326 mm^3^ (IQR: 0.135–1.091) at baseline to 0.283 mm^3^ (IQR: 0.089–0.806) at the final follow-up (median reduction: −0.045 mm^3^; 95% CI: −0.122 to −0.002; *p* = 0.034; Wilcoxon signed-rank test).

Regarding the relative response, we observed a median percentage reduction in IRF volume of 15.6% (IQR: −53.5% to +10.3%), corresponding to a small-to-moderate effect size (r = 0.27; *p* = 0.007). At the follow-up, complete resolution of SRF (0 mm^3^) was observed in all cases (100%) (*p* = 0.031; one-tailed Wilcoxon signed-rank test). No changes were detected in EZ and ELM at the follow-up (all *p* > 0.05). The median CST was 342 µm (IQR: 307–392) at baseline and 340 µm (IQR: 298–418) at the follow-up, with no statistical difference (*p* = 0.78) (Table 1).

### 3.8. Predictors of Anatomical Outcomes

No significant correlation was found between the reduction in IRF volume and the change in CST (ρ = 0.076; *p* = 0.55). Higher baseline IRF volume predicted greater absolute reduction (β = 0.36; *p* < 0.001; R^2^ = 0.44; Spearman ρ = 0.27; *p* = 0.029), whereas no association was found with %IRF reduction (ρ = −0.074; *p* = 0.56) or with baseline CST (ρ = 0.022; *p* = 0.86).

Baseline IRF volume did not differ between treatment-naïve and previously treated eyes (*p* = 0.33), but naïve eyes showed a significantly greater relative response (median %IRF reduction: 59.6% vs. 11.5%; *p* = 0.029). HRF counts and ELM/EZ integrity remained unchanged at follow-up (all *p* > 0.05).

**Figure 2:**
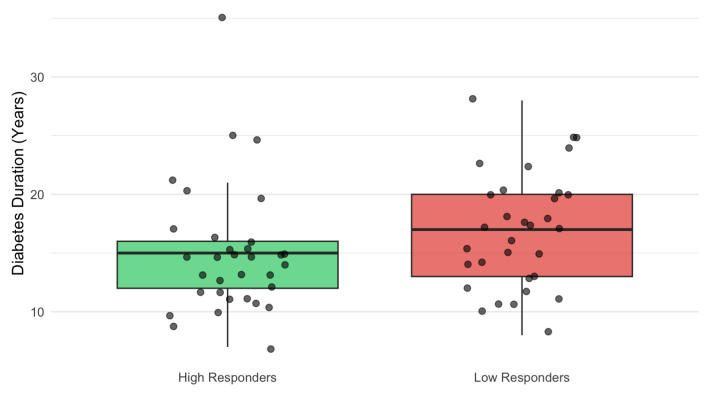
Impact of diabetes duration on anatomical response to SMPL. Boxplots showing the distribution of diabetes duration (in years) stratified by anatomical response (% IRF reduction). Patients classified as early high responders (median IRF reduction > median of the cohort, green box) had a significantly shorter disease duration compared to early low responders (red box). Black circles represent individual eyes. The central line indicates the median, box limits indicate the interquartile range, and whiskers extend to 1.5 times the interquartile range. The difference between groups was statistically significant (Wilcoxon rank-sum test, *p* = 0.025). SMPL = subthreshold micropulse laser; IRF = intraretinal fluid.

Stratification by median %IRF reduction identified early high responders (median −53.5%, IQR −72.9 to −36.5) and early low responders (median +10.3%, IQR −0.86 to +31.7). Early high responders had a shorter duration of diabetes (14.5 vs. 17 years; *p* = 0.025) (Figure 2). 

No significant differences were observed for age (*p* = 0.84), baseline BCVA (*p* = 0.17), baseline IRF volume (*p* = 0.89), IVT HbA1c (*p* = 0.07), number of prior IVTs (*p* = 0.23), or prior PRP (*p* = 0.21). Diabetes duration was not associated with baseline BCVA (ρ = 0.07; *p* = 0.58) or BCVA change (ρ = −0.08; *p* = 0.54).

### 3.9. Time–Response Relationship

Follow-up OCT was obtained at a median of 52 days after SMPL (IQR 41–60).

Longer follow-up duration correlated with greater absolute IRF reduction (ρ = −0.30, *p* = 0.014).

### 3.10. Impact of the Number of IVTs and HbA1c

When stratifying eyes by the median number of prior IVTs, neither absolute nor percentual IRF reduction differed significantly (ΔIRF *p* = 0.44; %IRF change *p* = 0.33). Regarding the HbA1c levels, %IRF change showed a trend (*p* = 0.063). To verify the factors associated with a better % IRF reduction, a multivariate linear regression analysis was performed, adjusting for age, diabetes duration, HbA1c, and baseline IRF volume. Treatment-naïve status emerged as the only significant independent predictor of percentage IRF reduction (β= 44.59; *p* = 0.028).

Specifically, being treatment-naïve was associated with an approximate 45% greater relative fluid reduction compared to pretreated eyes, independent of potential confounders. Diabetes duration showed a negative trend (β = −2.74), suggesting that efficacy may decrease by approximately 2.7% for each additional year of disease duration, although this did not reach statistical significance in the multivariate model (*p* = 0.085). Among pretreated eyes, no significant difference in the proportion of high versus early low responders was observed between those previously treated with anti-VEGF agents (n = 35) and those treated with intravitreal steroid implants (n = 16) (*p* = 1.0; Fisher’s exact test).

## 4. Discussion

In the present study, we aimed to identify anatomical and systemic predictors of early subthreshold micropulse laser (SMPL) response in center-involving diabetic macular edema (DME) using an AI-based approach. Our findings confirm that SMPL treatment is safe and effective, with improvement or stability of best-corrected visual acuity (BCVA) in 93.8% of eyes after a median of 52 days from treatment. Notably, no signs of laser-induced damage were reported. This safety profile is consistent with extensive literature, both in trials and real-world scenarios [38,39], which established that SMPL maintains visual function without the iatrogenic scarring associated with conventional photocoagulation [18].

From an anatomical perspective, our data showed that AI-driven biomarkers may reveal treatment effects and systemic associations that conventional metrics fail to capture. AI analysis of fluid compartments revealed distinct response patterns. We observed a reduction in absolute IRF (*p* = 0.034). In our cohort, preliminary observations on the subset of eyes with SRF (n = 5) showed complete resolution of subretinal fluid (SRF) in the affected cases (5/65) (*p* = 0.031).

By targeting the RPE, SMPL enhances the function of the outer blood–retinal barrier and upregulates RPE pump function, thereby facilitating the active transport of fluid from the subretinal space [40]. Previous studies have suggested that SRF presence is the direct consequence of rupture in the RPE barrier, which may be an acute rather than a chronic sign, compared with IRF, which involves Müller cell dysfunction and chronic DME [41]. For instance, to observe a significant improvement in Müller cells’ metabolic activity compared to baseline, it is necessary to undergo up to 12 months of SMPL treatment [15], highlighting that the effect on IRF may be slower compared to SRF. This is also supported by real-world evidence of SMPL in DME showing superior resolution rates for SRF (74%) compared to IRF (35%) after 3 months of treatment [27].

However, while the complete disappearance of SRF in this study (*p* = 0.031) is promising, it must be interpreted with caution, given the low baseline prevalence of SRF (5/65). This efficacy is further corroborated by the success of SMPL in diseases with a major SRF component, such as chronic central serous chorioretinopathy (cCSC), where it shows efficacy comparable to existing treatments but with an excellent safety profile [42].

In our cohort, no changes were detectable at the follow-up regarding outer retinal biomarkers (EZ and ELM). However, the effect of SMPL on these biomarkers may occur in a longer time interval, so it should be used cautiously.

Regarding quantitative response, SMPL induced a statistically significant median reduction in IRF percentage volume of 15.6% (*p* = 0.007), showing a small-to-moderate effect size (r = 0.27). Conversely, central subfield thickness (CST) remained stable (*p* = 0.78) and showed a negligible effect size (r = 0.03) in the early response phase. This discrepancy suggests that CST is not a sensitive biomarker for detecting early SMPL response, highlighting the value of AI-driven volumetric analysis. The literature on early CST response is controversial, with reports of stability at 1–2 months [22,39] and 4 months [18], followed by improvements at 6 and 12 months [19,29]. Consequently, reliance solely on CST variations may lead to the erroneous conclusion of early treatment failure.

The integration of AI-based volumetric metrics into clinical practice may refine decision-making algorithms. Particularly in the absence of CST changes, detection of a reduction in volumetric fluid would encourage clinicians to continue SMPL treatment rather than unnecessarily switching to anti-VEGF therapy. On the other hand, identifying non-responders at an earlier stage would allow for a prompt transition to the most effective treatment available.

Furthermore, our analysis challenges the reliance on baseline thickness as a predictor of outcome. Our data showed that patients with CST > 400 µm showed a favorable response to SMPL in terms of IRF % reduction. Baseline IRF volume did not correlate with the percentage of fluid resolution (*p* = 0.56), suggesting a proportional therapeutic effect, regardless of initial IRF severity. This contrasts with the seminal findings of Mansouri et al., who identified an arbitrary CST threshold (>400 µm) above which SMPL was considered ineffective [28]. However, it should be noted that the group “>400 µm” had a median thickness of 605 µm (IQR: 442–858), whereas our group had a median thickness of 342 µm (IQR: 307–392) with a maximum of 635 µm [28]. However, a recent study showed that patients with CST > 400 µm showed no improvement in BCVA or CST after 3 months. Nonetheless, in this cohort, only one eye was not previously treated, highlighting that the possible reduction in BCVA improvement was related to chronic DME rather than CST [43]. In fact, in our multivariate analysis, being treatment-naïve was the strongest independent predictor of fluid reduction, with naïve eyes achieving a median relative reduction of 59.6% compared to just 11.5% in pretreated eyes (*p* = 0.029). A potential confounder in the superior response of treatment-naïve eyes is the duration of the macular edema itself, which is typically shorter in naïve patients compared to the pretreated cohort. Although the retrospective nature of our study limits the precise determination of edema onset for every patient, our multivariate model explicitly adjusted for diabetes duration, serving as a proxy for systemic metabolic chronicity.

The finding that naïve status remained the strongest independent predictor, even after adjusting for systemic factors, suggests that the integrity of the RPE is the crucial substrate for SMPL efficacy. Pretreated eyes, often subjected to chronic fluid exposure and repeated intravitreal mechanics, may suffer from a ‘functional exhaustion’ of the RPE pump that limits the photo-stimulation response. This reinforces the clinical imperative of positioning SMPL as an early-intervention strategy in the therapeutic algorithm, rather than reserving it as a rescue therapy for recalcitrant, chronic cases. Further longitudinal studies considering naïve patients with CST > 400 compared with CST < 400 µm at baseline are needed to confirm our findings.

In our group, in the early phase of SMPL response, the percentage of AI-segmented IRF volume decreased significantly (*p* = 0.007), showing a small-to-moderate effect size (r = 0.27). However, we observed a dissociation between anatomical and functional outcomes: the percentage of IRF reduction did not statistically correlate with the magnitude of BCVA gain. This lack of linear correlation is expected in DME for several reasons. First, global volumetric reduction (measured over a 6 × 6 mm grid) does not necessarily reflect foveal status; a significant reduction in peripheral edema improves retinal health but may not immediately impact central acuity. Second, visual recovery depends on the integrity of foveal microstructures (ELM and EZ) [44,45,46], which were not affected by SMPL treatment in the early phase in our group (all *p* > 0.05). Third, SMPL may induce functional improvements before visible structural variations, as previously suggested [47].

Interestingly, we found that the magnitude of IRF reduction was significantly time-dependent: a longer interval between treatment and follow-up correlated with a greater percentage decrease in fluid (*p* = 0.027). This allows us to exclude a residual tail effect of previous anti-VEGF treatment. Anti-VEGF drugs and dexamethasone implants follow a known pharmacokinetic decay, with efficacy peaking early and waning over 4–8 weeks [48,49]. Conversely, we obtained a “crescendo” effect, consistent with the mechanism of sublethal RPE stimulation, which provides a progressive, long-term restoration of retinal homeostasis.

The AI-based analysis also allowed for the identification of correlations with systemic factors. Patients with a shorter duration of diabetes (14.5 vs. 17 years) were significantly more likely to be early high responders (*p* = 0.025) in terms of percentage IRF decrease. This, combined with the lower response in pretreated patients, confirms that chronic metabolic stress in long-standing diabetes may compromise the efficacy of the RPE response to SMPL. Interestingly, chronological age was almost identical between high and low responders, indicating that therapeutic efficacy is modulated by the duration of systemic disease rather than the patient’s biological age. Regarding HbA1c levels, our cohort demonstrated no correlation with baseline IRF volumes (*p* = 0.41). In a study by Işık et al., it was found that higher levels of HbA1c (>8%) are inversely related to the thickness of RPE. Interestingly, patients with thinner RPE had a higher probability of needing a second treatment with SMPL, and baseline retinal thickness was comparable between these two groups [30]. Crucially, the correlation of responsiveness to HbA1c levels is also controversial for anti-VEGF treatment [50,51,52]. However, our findings must be interpreted with caution, given the well-compensated nature of our cohort (median 6.4%, all ≤ 7.1%) compared with the population of this study. Therefore, further studies are needed to assess the potential impact of severe metabolic decompensation on early response to SMPL.

Finally, AI quantification may serve as a sensitive tool to assess responsiveness to SMPL compared with CST. Our study supports the theory that chronic remodeling in DME affects anatomical outcomes. However, longitudinal studies are required to confirm whether “low responders” are simply “slow responders.”

### Limitations and Future Directions

This study has several limitations. First, the retrospective cross-sectional design may introduce selection bias and limit the ability to establish causal relationships. Second, follow-up was not fixed, reflecting real-world practice. In addition, changes in visual acuity were analyzed only with BCVA, not including microperimetry, due to the real-world nature of the data. The sample size, while adequate for the primary analyses, limits the generalizability of multivariable models. More complex predictive modeling approaches would risk overfitting and were therefore not performed. Finally, our cohort was metabolically well controlled in the short term (HbA1c ≤ 7.1%), leading to potential underestimation of the HbA1c as a factor involved in the early response to SMPL treatment. Additionally, the exclusion of low-quality images for AI processing may have selected for patients with clearer media. However, the use of a validated deep learning model eliminates inter-observer bias in segmentation, providing a robust objective assessment of fluid dynamics.

## 5. Conclusions

The study indicates that the early response to SMPL treatment is accompanied by significant improvements in functional and anatomical factors. AI-driven analysis allowed for a better characterization of anatomical response compared with conventional metrics, which were less informative. Short-term response to SMPL may be influenced by the duration of the systemic disease and the chronicity of the local edema. In this context, we hypothesize that metabolic age could be more relevant than chronological age, affecting the capacity of RPE to react to SMPL stimulation. However, further studies with longitudinal follow-up are needed.

## Figures and Tables

**Table 1:** Anatomical and functional outcomes.

Outcome Measure	Baseline (T0) Median [IQR]	Follow-Up (T1) Median [IQR]	*p*-Value
BCVA (logMAR)	0.22 [0.15–0.30]	0.15 [0.10–0.30]	<0.001
Central Subfield Thickness (CST, µm)	342.00 [307–392]	340.00 [298–418]	0.78
Intraretinal Fluid (IRF, mm^3^)	0.326 [0.135–1.091]	0.283 [0.089–0.806]	0.034
Subretinal fluid (SRF, mm^3^)	0.026 mm^3^; [0.020–0.046]	0.00 mm^3^ [0–0]	0.031

## Data Availability

The original contributions presented in this study are included in the article. Further inquiries can be directed to the corresponding author.

## Abbreviations

The following abbreviations are used in this manuscript:

AI	Artificial intelligence
BCVA	Best-corrected visual acuity
cCSC	Chronic central serous chorioretinopathy
CE	Conformité Européenne (European conformity marking)
CST	Central subfield thickness
DL	Deep learning
DME	Diabetic macular edema
ETDRS	Early Treatment Diabetic Retinopathy Study
EZ	Ellipsoid zone
FAZ	Foveal avascular zone
HbA1c	Hemoglobin A1c
HRF	Hyperreflective foci
IRF	Intraretinal fluid
IVT	Intravitreal therapy
OCT	Optical coherence tomography
PRP	Panretinal photocoagulation
RPE	Retinal pigment epithelium
SD-OCT	Spectral-domain optical coherence tomography
SMPL	Subthreshold micropulse laser
SRF	Subretinal fluid

## References

[1] Daruich A., Matet A., Moulin A., Kowalczuk L., Nicolas M., Sellam A., Rothschild P.R., Omri S., Gélizé E., Jonet L. (2018). Mechanisms of macular edema: Beyond the surface. Prog. Retin. Eye Res..

[2] Gonder J.R., Walker V.M., Barbeau M., Zaour N., Zachau B.H., Hartje J.R., Li R. (2014). Costs and Quality of Life in Diabetic Macular Edema: Canadian Burden of Diabetic Macular Edema Observational Study (C-REALITY). J. Ophthalmol..

[3] Schmidt-Erfurth U., Garcia-Arumi J., Bandello F., Berg K., Chakravarthy U., Gerendas B.S., Jonas J., Larsen M., Tadayoni R., Loewenstein A. (2017). Guidelines for the Management of Diabetic Macular Edema by the European Society of Retina Specialists (EURETINA). Ophthalmologica.

[4] National Institute for Health and Care Excellence (NICE) (2024). Diabetic Retinopathy: Management and Monitoring.

[5] Parravano M., Cennamo G., Di Antonio L., Grassi M.O., Lupidi M., Rispoli M., Savastano M.C., Veritti D., Vujosevic S. (2024). Multimodal imaging in diabetic retinopathy and macular edema: An update about biomarkers. Surv. Ophthalmol..

[6] Khoramnia R., Nguyen Q.D., Kertes P.J., Sararols Ramsay L., Vujosevic S., Anderesi M., Igwe F., Eter N. (2024). Exploring the role of retinal fluid as a biomarker for the management of diabetic macular oedema. Eye.

[7] Couturier A., Wykoff C.C., Lupidi M., Udaondo P., Peto T., Pintard P.J. (2025). Anatomic biomarkers as potential endpoints in diabetic macular edema: A systematic literature review with identification of macular volume as a key surrogate for visual acuity. Surv. Ophthalmol..

[8] Midena E., Lupidi M., Toto L., Covello G., Veritti D., Pilotto E., Cicinelli M.V., Lattanzio R., Figus M., Midena G. (2025). AI-Assisted OCT Clinical Phenotypes of Diabetic Macular Edema: A Large Cohort Clustering Study. J. Clin. Med..

[9] Spooner K.L., Guinan G., Koller S., Hong T., Chang A.A. (2019). Burden Of Treatment Among Patients Undergoing Intravitreal Injections For Diabetic Macular Oedema In Australia. Diabetes Metab. Syndr. Obes..

[10] Choi K., Park S.J., Yoon H., Choi S., Mun Y., Kim S., Yoo S., Woo S.J., Park K.H., Na J. (2024). Patient-Centered Economic Burden of Diabetic Macular Edema: Retrospective Cohort Study. JMIR Public Health Surveill..

[11] Tabano D., Watane A., Gale R., Cox O., Hill S.R., Longworth L., Oluboyede Y., Ahmed A., Patel N.A. (2025). The Economic Burden of Anti-Vascular Endothelial Growth Factor on Patients and Caregivers in the UK, Europe, and North America. Ophthalmol. Ther..

[12] Edington M., Connolly J., Chong N.V. (2017). Pharmacokinetics of intravitreal anti-VEGF drugs in vitrectomized versus non-vitrectomized eyes. Expert Opin. Drug Metab. Toxicol..

[13] Israilevich R.N., Mansour H., Patel S.N., Garg S.J., Klufas M.A., Yonekawa Y., Regillo C.D., Hsu J. (2024). Risk of Endophthalmitis Based on Cumulative Number of Anti-VEGF Intravitreal Injections. Ophthalmology.

[14] Zafar S., Walder A., Virani S., Biggerstaff K., Orengo-Nania S., Chang J., Channa R. (2023). Systemic Adverse Events Among Patients With Diabetes Treated With Intravitreal Anti-Vascular Endothelial Growth Factor Injections. JAMA Ophthalmol..

[15] Midena E., Micera A., Frizziero L., Pilotto E., Esposito G., Bini S. (2019). Sub-threshold micropulse laser treatment reduces inflammatory biomarkers in aqueous humour of diabetic patients with macular edema. Sci. Rep..

[16] Sabal B., Wylęgała E., Teper S. (2025). Impact of Subthreshold Micropulse Laser on the Vascular Network in Diabetic Macular Edema: An Optical Coherence Tomography Angiography Study. Biomedicines.

[17] Vujosevic S., Lupidi M., Donati S., Astarita C., Gallinaro V., Pilotto E. (2024). Role of inflammation in diabetic macular edema and neovascular age-related macular degeneration. Surv Ophthalmol..

[18] Fazel F., Bagheri M., Golabchi K., Jahanbani Ardakani H. (2016). Comparison of subthreshold diode laser micropulse therapy versus conventional photocoagulation laser therapy as primary treatment of diabetic macular edema. J. Curr. Ophthalmol..

[19] Kikushima W., Furuhata Y., Shijo T., Matsumoto M., Sakurada Y., Viel Tsuru D., Kashiwagi K. (2025). Comparison of one-year real-world outcomes between red (670 nm) subthreshold micropulse laser treatment and intravitreal aflibercept injection for treatment-naïve diabetic macular edema. Photodiagn. Photodyn. Ther..

[20] Jiang Y., He W., Qi S. (2025). Evaluating the efficacy of subthreshold micropulse laser combined with anti-VEGF drugs in the treatment of diabetic macular edema: A systematic review and meta-analysis. Front. Endocrinol..

[21] Vujosevic S., Toma C., Villani E., Brambilla M., Torti E., Leporati F., Muraca A., Nucci P., De Cilla S. (2020). Subthreshold Micropulse Laser in Diabetic Macular Edema: 1-Year Improvement in OCT/OCT-Angiography Biomarkers. Transl. Vis. Sci. Technol..

[22] Ueda K., Shiraya T., Araki F., Hashimoto Y., Yamamoto M., Yamanari M., Ueta T., Minami T., Aoki N., Sugiyama S. (2021). Changes in entropy on polarized-sensitive optical coherence tomography images after therapeutic subthreshold micropulse laser for diabetic macular edema: A pilot study. PLoS ONE.

[23] Kirik F., Ersoz M.G., Atalay F., Akbulut E., Kucuk M., Koytak A., Ozdemir H. (2025). Outer Retinal Layer Deterioration Patterns in Eyes with Diabetic Serous Macular Detachment. Retina.

[24] Kessler L.J., Auffarth G.U., Bagautdinov D., Khoramnia R. (2021). Ellipsoid Zone Integrity and Visual Acuity Changes during Diabetic Macular Edema Therapy: A Longitudinal Study. J. Diabetes Res..

[25] Muftuoglu I.K., Mendoza N., Gaber R., Alam M., You Q., Freeman W.R. (2017). Integrity of Outer Retinal Layers after Resolution of Central Involved Diabetic Macular Edema. Retina.

[26] Tang L., Luo D., Qiu Q., Xu G.T., Zhang J. (2023). Hyperreflective Foci in Diabetic Macular Edema with Subretinal Fluid: Association with Visual Outcomes after Anti-VEGF Treatment. Ophthalmic Res..

[27] Passos R.M., Malerbi F.K., Rocha M., Maia M., Farah M.E. (2021). Real-life outcomes of subthreshold laser therapy for diabetic macular edema. Int. J. Retin. Vitr..

[28] Mansouri A., Sampat K.M., Malik K.J., Steiner J.N., Glaser B.M. (2014). Efficacy of subthreshold micropulse laser in the treatment of diabetic macular edema is influenced by pre-treatment central foveal thickness. Eye.

[29] Citirik M. (2019). The impact of central foveal thickness on the efficacy of subthreshold micropulse yellow laser photocoagulation in diabetic macular edema. Lasers Med. Sci..

[30] Işık M.U., Değirmenci M.F.K., Sağlık A. (2022). Factors affecting the response to subthreshold micropulse laser therapy used in center-involved diabetic macular edema. Lasers Med. Sci..

[31] Parravano M., Costanzo E., Querques G. (2020). Profile of non-responder and late responder patients treated for diabetic macular edema: Systemic and ocular factors. Acta Diabetol..

[32] Lang A., Carass A., Hauser M., Sotirchos E.S., Calabresi P.A., Ying H.S., Prince J.L. (2013). Retinal layer segmentation of macular OCT images using boundary classification. Biomed. Opt. Express.

[33] Roberts P.K., Vogl W.D., Gerendas B.S., Glassman A.R., Bogunovic H., Jampol L.M., Schmidt-Erfurth U.M. (2020). Quantification of Fluid Resolution and Visual Acuity Gain in Patients With Diabetic Macular Edema Using Deep Learning: A Post Hoc Analysis of a Randomized Clinical Trial. JAMA Ophthalmol..

[34] Cicinelli M.V., Leonardo B., Maiucci G., Martino G., Ziafati M., Bousyf S., Frizziero L., Lattanzio R., Midena E., Bandello F. (2025). What Lies beneath Diabetic Macular Edema: Latent Phenotypic Clustering and Differential Treatment Responses to Intravitreal Therapies. Ophthalmol. Sci..

[35] Midena E., Toto L., Frizziero L., Covello G., Torresin T., Midena G., Danieli L., Pilotto E., Figus M., Mariotti C. (2023). Validation of an Automated Artificial Intelligence Algorithm for the Quantification of Major OCT Parameters in Diabetic Macular Edema. J. Clin. Med..

[36] Chhablani J., SOLS (Subthreshold Laser Ophthalmic Society) Writing Committee (2022). Subthreshold Laser Therapy Guidelines for Retinal Diseases. Eye.

[37] Keunen J.E.E., Battaglia-Parodi M., Vujosevic S., Luttrull J.K. (2020). International Retinal Laser Society Guidelines for Subthreshold Laser Treatment. Transl. Vis. Sci. Technol..

[38] Frizziero L., Calciati A., Torresin T., Midena G., Parrozzani R., Pilotto E., Midena E. (2021). Diabetic Macular Edema Treated with 577-nm Subthreshold Micropulse Laser: A Real-Life, Long-Term Study. J. Pers. Med..

[39] Kikushima W., Shijo T., Furuhata Y., Sakurada Y., Kashiwagi K. (2021). Comparison of the 1-Year Visual and Anatomical Outcomes between Subthreshold Red (670 nm) and Yellow (577 nm) Micro-Pulse Laser Treatment for Diabetic Macular Edema. Pharmaceuticals.

[40] Bodea F., Bungau S.G., Bogdan M.A., Vesa C.M., Radu A., Tarce A.G., Purza A.L., Tit D.M., Bustea C., Radu A.F. (2023). Micropulse Laser Therapy as an Integral Part of Eye Disease Management. Medicina.

[41] Lai D., Wu Y., Shao C., Qiu Q. (2023). The Role of Müller Cells in Diabetic Macular Edema. Invest. Ophthalmol. Vis. Sci..

[42] Li X., Long H., Hu Q. (2022). Efficacy of Subthreshold Micropulse Laser for Chronic Central Serous Chorioretinopathy: A Meta-Analysis. Photodiagnosis Photodyn. Ther..

[43] Kim M., Park Y.G., Jeon S.H., Choi S.Y., Roh Y.J. (2020). The Efficacy of Selective Retina Therapy for Diabetic Macular Edema Based on Pretreatment Central Foveal Thickness. Lasers Med. Sci..

[44] Otani T., Yamaguchi Y., Kishi S. (2010). Correlation between Visual Acuity and Foveal Microstructural Changes in Diabetic Macular Edema. Retina.

[45] Ito S., Miyamoto N., Ishida K., Kurimoto Y. (2013). Association between External Limiting Membrane Status and Visual Acuity in Diabetic Macular Oedema. Br. J. Ophthalmol..

[46] Achiron A., Kydyrbaeva A., Man V., Lagstein O., Burgansky Z., Blumenfeld O., Bar A., Bartov E. (2017). Photoreceptor Integrity Predicts Response to Anti-VEGF Treatment. Ophthalmic Res..

[47] Vujosevic S., Frizziero L., Martini F., Bini S., Convento E., Cavarzeran F., Midena E. (2018). Single Retinal Layer Changes After Subthreshold Micropulse Yellow Laser in Diabetic Macular Edema. Ophthalmic Surg. Lasers Imaging Retin..

[48] Eissing T., Stewart M.W., Qian C.X., Rittenhouse K.D. (2021). Durability of VEGF Suppression with Intravitreal Aflibercept and Brolucizumab: Using Pharmacokinetic Modeling to Understand Clinical Outcomes. Transl. Vis. Sci. Technol..

[49] Won J., Kang J., Kang W. (2024). Comparative Ocular Pharmacokinetics of Dexamethasone Implants in Rabbits. J. Ocul. Pharmacol. Ther..

[50] Singh R.P., Habbu K., Ehlers J.P., Lansang M.C., Hill L., Stoilov I. (2016). The Impact of Systemic Factors on Clinical Response to Ranibizumab for Diabetic Macular Edema. Ophthalmology.

[51] Brown D.M., Schmidt-Erfurth U., Do D.V., Holz F.G., Boyer D.S., Midena E., Heier J.S., Terasaki H., Kaiser P.K., Marcus D.M. (2015). Intravitreal Aflibercept for Diabetic Macular Edema: 100-Week Results from the VISTA and VIVID Studies. Ophthalmology.

[52] Bressler S.B., Odia I., Maguire M.G., Dhoot D.S., Glassman A.R., Jampol L.M., Marcus D.M., Solomon S.D., Sun J.K. (2019). Diabetic Retinopathy Clinical Research Network. Factors Associated With Visual Acuity and Central Subfield Thickness Changes When Treating Diabetic Macular Edema With Anti-Vascular Endothelial Growth Factor Therapy: An Exploratory Analysis of the Protocol T Randomized Clinical Trial. JAMA Ophthalmol..

